# Supplementary material for: Analysis of the differences in physicochemical properties, volatile compounds, and microbial community structure of pit mud in different time spaces
Source: PeerJ. 2024 Feb 29;12:e17000. doi: 10.7717/peerj.17000 (PMC10909342; doi:10.7717/peerj.17000)
Supplement: Supplemental Information 1 [file peerj-12-17000-s001.docx]

The HiSeq sequencing data are available at the Sequence Read Archive (SRA) of NCBI: PRJNA1010531. https://submit.ncbi.nlm.nih.gov/subs/sra/.
