# Supplementary material for: Analysis of the differences in physicochemical properties, volatile compounds, and microbial community structure of pit mud in different time spaces
Source: PeerJ. 2024 Feb 29;12:e17000. doi: 10.7717/peerj.17000 (PMC10909342; doi:10.7717/peerj.17000)
Supplement: Supplemental Information 3 [file peerj-12-17000-s003.docx]

**Figure S1**

PM sampling location


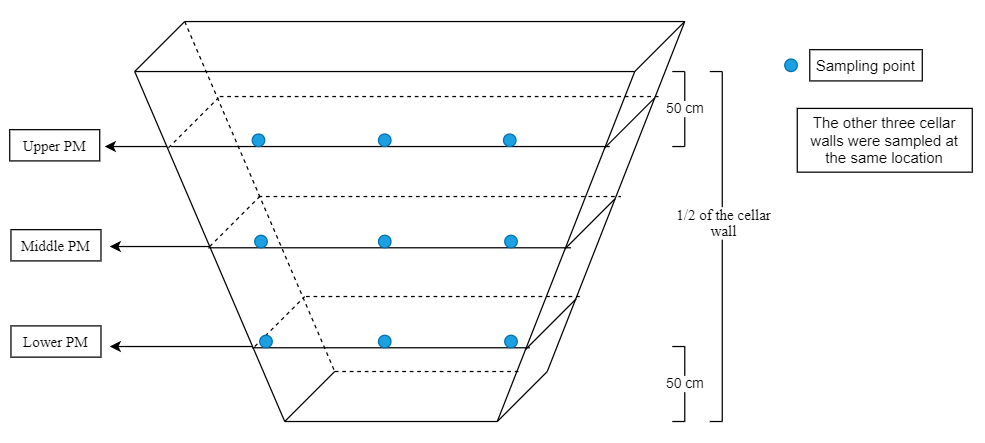


**Figure S2**

Sobs dilution curve of bacteria(A) and archaea(B)


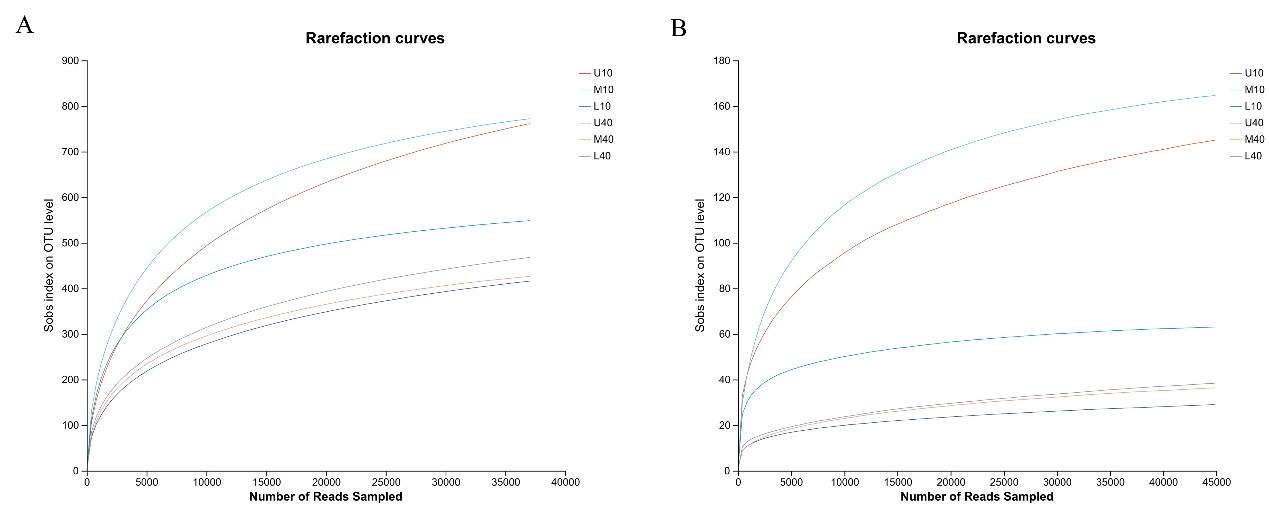


**Table S1:**

Bacterial microbial community Alpha diversity index（A）

| Sample\Estimators | Sobs | Shannon | Ace | Chao1 | Coverage |
| --- | --- | --- | --- | --- | --- |
| U10 | 761.67±32.93^a^ | 4.12±0.14^ab^ | 951.11±69.70^a^ | 926.60±41.07^a^ | 99.46% |
| M10 | 772.33±108.31^a^ | 4.72±0.05^a^ | 851.30±139.47^a^ | 852.60±135.51^a^ | 99.66% |
| L10 | 548.67±138.93^b^ | 4.32±0.20^ab^ | 599.43±186.23^b^ | 605.60±174.01^b^ | 99.79% |
| U40 | 416.00±53.11^b^ | 3.32±0.77^c^ | 531.07±44.84^b^ | 524.88±39.48^b^ | 99.70% |
| M40 | 426.33±25.48^b^ | 3.09±0.42^c^ | 509.29±35.36^b^ | 497.73±39.36^b^ | 99.75% |
| L40 | 468.00±34.60^b^ | 3.65±0.20^bc^ | 587.03±59.48^b^ | 573.29±29.97^b^ | 99.68% |

Sobs: reflects the changes in the population of each species

Shannon: reflects the diversity of the microbial community

Ace and Chao1: reflect the richness of microbial community

**Table S1:**

Archaeal microbial community Alpha diversity index（B）

| Sample\Estimators | Sobs | Shannon | Ace | Chao1 | Coverage |
| --- | --- | --- | --- | --- | --- |
| U10 | 145.00±36.51^ab^ | 2.64±0.05^a^ | 185.74±44.31^a^ | 178.80±39.81^a^ | 99.92% |
| M10 | 164.67±102.89^a^ | 2.35±0.17^a^ | 179.80±98.83^a^ | 174.74±99.17^a^ | 99.95% |
| L10 | 63.00±27.62^bc^ | 1.99±1.05^ab^ | 66.95±26.77^b^ | 64.61±26.75^b^ | 99.99% |
| U40 | 29.00±6.56^c^ | 1.27±0.08^bc^ | 39.92±9.91^b^ | 36.97±10.66^b^ | 99.98% |
| M40 | 36.33±24.58^c^ | 0.65±0.48^c^ | 56.30±28.94^b^ | 43.50±27.83^b^ | 99.98% |
| L40 | 38.33±17.16^c^ | 1.23±0.28^bc^ | 62.98±23.59^b^ | 59.44±26.40^b^ | 99.97% |

Sobs: reflects the changes in the population of each species

Shannon: reflects the diversity of the microbial community

Ace and Chao1: reflect the richness of microbial community

**Table S2:**

The content of volatile compounds in different samples

| Volatile compounds(μg/g)\ Sample | U10 | M10 | L10 | U40 | M40 | L40 |
| --- | --- | --- | --- | --- | --- | --- |
| Ethyl butyrate | 0 | 0 | 3.83 | 0 | 0 | 0.09 |
| Ethyl pentanoate | 0 | 1.85 | 3.09 | 0 | 3.4 | 11.74 |
| Ethyl caproate | 11.65 | 36.98 | 51.79 | 28.33 | 286.67 | 1053.54 |
| Hexyl acetate | 0 | 0 | 0 | 0 | 0 | 3.36 |
| Propyl caproate | 0 | 0 | 0 | 0 | 5.8 | 24.97 |
| Ethyl heptanoate | 0 | 2.68 | 2.44 | 1.65 | 27.52 | 67.5 |
| Ethyl lactate | 0 | 0 | 0 | 0 | 4.42 | 16.3 |
| Isobutyl hexanoate | 0 | 0 | 0 | 0 | 0 | 3.21 |
| Butyl hexanoate | 0 | 0.28 | 0.56 | 0.18 | 7.42 | 20.38 |
| Ethyl caprylate | 4.17 | 6.12 | 4.04 | 5.32 | 53.12 | 138.96 |
| Isoamyl hexanoate | 0 | 0 | 0 | 0 | 2.99 | 6.36 |
| Pentyl hexanoate | 0 | 0 | 0 | 0 | 4.97 | 14.13 |
| Propyl caprylate | 0 | 0 | 0 | 0 | 0 | 2.83 |
| Ethyl nonanoate | 0 | 0 | 0 | 0 | 0.65 | 1.6 |
| Hexyl caproate | 0.93 | 0 | 0 | 3.59 | 46.34 | 171.94 |
| Ethyl decanoate | 0 | 0 | 0 | 0 | 2.8 | 4.61 |
| Octyl heptanoate | 0 | 0 | 0 | 0 | 0 | 16.44 |
| Hexyl caprylate | 0 | 0 | 0 | 0 | 0 | 28.46 |
| Ethyl hexadecanoate | 6.54 | 24.63 | 26.34 | 2.54 | 11.64 | 0 |
| Acetic acid | 2.87 | 4.56 | 5.01 | 1.61 | 9.29 | 9.37 |
| Propionic acid | 0 | 0 | 0 | 0 | 0.76 | 0.67 |
| 2-Methylpropionic acid | 0 | 0 | 0 | 0 | 1.07 | 2.92 |
| Isobutyric acid | 1.85 | 0 | 0 | 0 | 0 | 0 |
| Butyric acid | 17.33 | 8.11 | 9.94 | 34.61 | 13.95 | 26.9 |
| 3-Methylbutyric acid | 0 | 0 | 0 | 0 | 2.69 | 7.87 |
| Valeric acid | 2.15 | 1.53 | 2.53 | 0 | 9.22 | 21.49 |
| Hexanoic acid | 31.46 | 18.94 | 34.53 | 4.76 | 371.81 | 1669.48 |
| Heptanoic acid | 1.59 | 1.66 | 1.44 | 0 | 40.28 | 226.78 |
| Octanoic acid | 18.16 | 6.73 | 4.45 | 2.13 | 115.7 | 727.15 |
| Decanoic acid | 0 | 0 | 0 | 0 | 0 | 45.96 |
| 2-Ethyl-1-hexanol | 1.42 | 0 | 0 | 0 | 0 | 0 |
| Cyclohexanol | 0 | 3.56 | 2.64 | 0 | 0 | 0 |
| n-Hexanol | 0 | 0 | 0 | 3.04 | 3.99 | 46.00 |
| Heptanol | 0 | 0 | 0 | 0 | 0 | 3.39 |
| 2-Octanone | 0 | 0 | 0 | 4.35 | 0 | 0 |
| Phenol | 0 | 0 | 0 | 0 | 0 | 4.22 |
| 4-Methylphenol | 0 | 0 | 0 | 1.46 | 6.92 | 16.61 |
| 2,4-Di-tert-butylphenol | 44.72 | 19.99 | 18.07 | 14.19 | 8.67 | 22.74 |
| Ethyl phenylacetate | 0 | 0 | 0 | 0 | 0 | 2.46 |
| Ethyl phenylpropionate | 0 | 0 | 0 | 0 | 2.95 | 4.72 |
